# Facilitating Collective Psychosocial Resilience in the Public in Emergencies: Twelve Recommendations Based on the Social Identity Approach

**DOI:** 10.3389/fpubh.2019.00141

**Published:** 2019-06-04

**Authors:** John Drury, Holly Carter, Chris Cocking, Evangelos Ntontis, Selin Tekin Guven, Richard Amlôt

**Affiliations:** ^1^School of Psychology, University of Sussex, Brighton, United Kingdom; ^2^Emergency Response Department Science and Technology, Health Protection Directorate, Public Health England, Salisbury, United Kingdom; ^3^School of Health Sciences, University of Brighton, Brighton, United Kingdom; ^4^School of Psychology, Politics, and Sociology, Canterbury Christ Church University, Canterbury, United Kingdom

**Keywords:** collective resilience, social identity, crowds, emergency, disaster, guidance

## Abstract

Accumulated evidence demonstrates the centrality of social psychology to the behavior of members of the public as immediate responders in emergencies. Such public behavior is a function of social psychological processes—in particular *identities and norms*. In addition, what the authorities and relevant professional groups *assume* about the social psychology of people in emergencies shapes policy and practice in preparedness, response, and recovery. These assumptions therefore have consequences for the public's ability to act as immediate responders. In this Policy and Practice Review, we will do three things. First, we will overview research on the behavior of survivors of emergencies and disasters, drawing out key factors known to explain the extent to which survivors cooperate in these events and contribute to safe collective outcomes. We will demonstrate the utility of the social identity approach as an overarching framework for explaining the major mechanisms of collective supportive behavior among survivors in emergencies. Second, we will critically review recent and current UK government agency guidance on emergency response, focusing particularly on what is stated about the role of survivors in emergencies and disasters. This review will suggest that the “community resilience” agenda has only been partly realized in practice, but that the social identity approach is progressing this. Third, we will derive from the research literature and from dialogue with groups involved in emergencies a set of 12 recommendations for both emergency managers and members of the public affected by emergencies and disasters. These focus on the crucial need to build shared identity and to communicate, and the connection between these two aims. Including our recommendations within emergency guidance and training will facilitate *collective psychosocial resilience*, which refers to the way a shared identity allows groups of survivors to express and expect solidarity and cohesion, and thereby to coordinate and draw upon collective sources of support. In sum, this evidence-base and the recommendations we derive from it will help professionals involved in emergency management to support public resilient behaviors and will help the public to develop and maintain their own capacity for such resilience.

## Introduction

Social psychology is critical to the behavior of members of the public as immediate responders in emergencies, in three senses. First, such public behavior is a function of *group processes*—norms, relationships, and social identities. Second, what the authorities and professional groups *assume* about the social psychology of people in emergencies shapes policy and practice in preparedness, response, and recovery. Third, these policies and practices in turn impact upon the public's ability to act as immediate responders. The “disaster myths” of mass panic, public helplessness, and inevitable disorder have been criticized not only for being distortions of how survivors actually behave, but also for rationalizing emergency management strategies that undermine the public's capacity for resilient behaviors (1, 2). Recommendations based on assumptions of inherent collective vulnerability in the public can serve to *create* this very vulnerability (3): for example, the imperative “don't tell them—they'll only panic” leads response agencies to restrict information (4, 5). Providing information increases efficacy in the public (6); in contrast, a perceived lack of information provision increases public anxiety and distress (4, 7, 8).

Decades of research on collective behavior in emergencies and disasters has shown that survivors often provide each other with social support—both practical and emotional. Indeed, most lives are saved by the “average” citizen, whether “bystander” or fellow survivor, rather than by the professionals (9). Survivors have been dubbed “professional and civilian first responders” (10), “zero responders” (11–13), and “the fourth emergency service” (14). They are typically willing to help, even if they don't have specialist skills. This mutual aid among survivors arises endogenously from an interaction between social psychological factors and features of the environment; but authority and responder actions can support or inhibit the process, depending on their awareness of how it works.

Since the 9/11 attacks on the World Trade Center, UK guidance on emergencies has reflected an increased acknowledgment of this public capacity for resilient behaviors, particularly at the community level, as well as greater recognition of the *need* for such public involvement. However, recent critical analyses (2, 15–17) have argued that: some of the guidance also draws upon older models of public behavior in emergencies according to which the public is inherently psychologically vulnerable and uncooperative; conceptions of the social group are typically underdeveloped or unexamined in the guidance; therefore some of the practical recommendations in the guidance conflict with both the principle and the evidence of effective public participation in emergencies.

Given the importance of assumptions about social psychology in emergencies, there is a need among both policymakers and practitioners for an evidence-based theoretical framework that: makes sense of the widespread findings of public collective resilience; and is generative enough to enable practical implications to be derived. Such a framework can provide consistency and coherence to preparedness, response, and recovery, and can enhance operations in each of these areas, thereby contributing to saving lives. The social identity approach in social psychology has increasingly begun to play this role in civil contingencies planning, crowd safety management, and post-disaster psychosocial care, and is therefore the focus of this article.

The social identity approach (18, 19) began as an academic theory of intergroup relations, and has been developed into a set of principles for a range of applied settings including organizational psychology, leadership, health behavior, clinical psychology, and policing (20, 21). In the area of emergencies and disasters, some of the recommendations deriving from the social identity approach echo those derived from other frameworks (or from practitioners' experience)—in particular the value of providing information and communication to enhance effective citizen response (9, 22). But the social identity approach provides a new rationale and new benefits for these good practices which had not been suggested previously. A core principle for all social identity based recommendations is that shared identity provides individuals with strengths and abilities that they do not have alone, and therefore that the role of the authorities is to support or facilitate that shared identity. This can sometimes include simply not getting in the way of the identity-based action of crowd members.

This Policy and Practice Review will first summarize existing research on the behavior of survivors in emergencies and disasters, drawing out key factors known to explain the extent to which survivors cooperate in these events and contribute to safe outcomes for others. It will show how an early emphasis in the literature on collective psychological vulnerability gave way to theories focused more on adaptive sociality; and it will suggest how the social identity approach provides an overarching explanatory framework for the key concepts found across the literature. Second, it will critically review what UK government agency guidance says about survivors' behaviors in emergencies and disasters, comparing this with the research evidence. Third, the article will specify 12 evidence-based and actionable policy and practice recommendations. These will help professional groups support public resilient behaviors as well as help the public to develop and maintain their own capacity for such resilience.

## From “Disaster Myths”^1^ to Models of Adaptive Sociality

The focus of the present article is those people directly affected by emergencies and disasters—“victims” themselves, survivors, and those under threat. While the phenomenon of “convergence” is well-established (24) and indicates the critical role of non-affected bystanders as immediate responders during emergencies, there are two reasons for our focus on those directly affected. The first is practical and the second is theoretical. Practically, survivors and those threatened by the incident are close enough to respond before anyone else. Therefore, they are particularly important, and there is a need to understand and support their actions. Theoretically, from the point of view of the individualistic assumptions that dominate both academic theory (25, 26) and everyday discourse (at least in the West), the behavior of these survivors seems a puzzle: the idea that people act as responders for others (often strangers) in incidents where their personal self-interest is threatened requires explanation. In the earliest accounts of human behavior in emergencies and disasters, this puzzle was not properly investigated and instead commentators drew on models which depicted behavior in emergencies as a collective pathology due to survivors' immersion in crowds.

Crowd psychology has thus been central to understandings (and misunderstandings) of public responses to emergencies and disasters. Yet there are a range of different crowd psychology theories. Some of those most deeply rooted in public (and professional) consciousness are poorly evidenced. More recent crowd psychology theories are grounded in extensive social scientific research, including not just observation but also interview, questionnaire survey, and experimental studies.

### “Mass Panic” and Other Collective Pathological Responses

The earliest view of public behavior in emergencies and disasters was that people in such events are prone to “panic,” meaning impulsive, selfish, and uncoordinated responses. In this account, the crowd is the conduit for this impulsivity and irrationality, via “contagion” (27, 28). Mass panic is said to occur when a crowd has only limited opportunity for escape from impending danger (29). “Panic” supposedly explains the high numbers of avoidable fatalities in emergency evacuations (30).

A second pathological psychological reaction said to occur in emergencies and disasters is helplessness, or “disaster syndrome” (31), which suggests that survivors are too stunned and passive to care for themselves (9, 32). While there is evidence that 15 per cent of people “freeze” in emergencies (33), the suggestion of collective helplessness is a stronger claim that passivity is a generic response among survivors.

Third, there is the suggestion that civil disorder is inevitable in emergencies and disasters. In this account, in emergencies the crowd operates as a “cloak” under which willful and uncontrolled criminality can take place; emergencies and disasters “bring out the worst in people” [(34), p. 556], especially antisocial behavior (35), rioting, and “looting”^2^ (9, 38, 39).

#### Critique of the “Collective Pathology” Approach

In the disasters literature, these three claims about collective response are called “disaster myths” because of the weight of evidence against them (39–41). “Panic” as a claim about default behaviors in an emergency has three problems. First, there is the problem of determining whether survivors' reactions are unreasonable within an event where there is often limited information (30, 40, 42, 43). In many cases, “panic” seems to be a *post-hoc* judgement rather than an explanation of process. Second, a number of detailed case studies (44–46) and reviews of the literature (47–49) conclude that panic is “rare.” Third, and most damning for the predictions of “panic,” but also for the disaster myths of helplessness and disorder, is the consistent evidence across different kinds of emergencies and disasters that those affected often help each other and cooperate. This is not to say that everyone cooperates or that all emergencies display equal degrees of help; some emergency evacuations are characterized by individualistic behavior and hence lack of coordination (30, 50, 51). Nevertheless there is a wealth of evidence that survivors commonly give each other support in fires (52, 53), earthquakes (54), hurricanes (55), and many other kinds of emergencies (56). As well as acting to support other survivors, there is evidence that survivors can be proactive in preventing further danger—for example confronting attackers in terrorist incidents (57).

#### Pervasiveness and Practical Implications of Disaster Myths

Despite the evidence against it, “mass panic” as a way of characterizing behavior in emergencies is found in everyday talk (42), news reporting (58), computer simulations (59), and in the views of professional responders (1, 38, 39). “Panic” is also evident in some official guidance, as we shall discuss.

This pervasiveness of “mass panic” and other “disaster myths” is not just an academic matter. Critics have argued that these notions have operated as rationales for inappropriate, inefficient, and even dangerous forms of emergency management (31, 35, 60). Thus, the “panic” and “helplessness” myths are said to be behind the ethos of mistrust in post-9/11 homeland security policies in the United States (61, 62). Fear that the public will panic has led to the withholding of information (4, 5) and is also the reason that event stewards use code words to communicate with each other that there is a fire or other threat (42, 63). Likewise, overstating of the prevalence of “looting,” and indeed more generally perceiving survivors' actions as “disorder,” has been shown to be highly consequential. For example, following Hurricane Katrina these beliefs were used to justify a coercive rather than humanitarian response, resulting in many more deaths (36, 37).

### Models of Sociality in Emergencies and Disasters

Within academic research, the accumulated evidence of survivors acting as responders prompted new kinds of explanations and models, superseding the “disaster myths.” Three kinds of explanations for mutual aid among survivors in emergencies have been proposed: emergent “disaster communities”; social norms; and existing social relationships. Here we review the evidence and argue that these three processes can to a large degree each be understood in terms of our perception of ourselves as group members, or *shared social identity*. We outline the social identity approach and describe how it has been applied to mass emergency behavior.

#### Emergent Disaster Communities

A number of researchers have suggested that the basis of widespread helping in emergencies and disasters is due to the emergence of a new social group among survivors, variously called a “community of sufferers” (47), “therapeutic community” (47), “altruistic community” (64), or “disaster community” (65). The existence and function of these groups has mostly been documented in the recovery phase (66–69), though there is also extensive evidence that they arise in the acute phase of emergencies (70, 71). Most of the work on them is sociological, and where psychological mechanisms are discussed, a suggestion is that disaster communities arise from perceptions of common fate, whereby the shared experience of disaster causes previous social group boundaries to dissolve (47, 71, 72).

#### Social Norms

A second explanation for supportive behavior among survivors is in terms of social norms—that is, rules or guides to conduct based on shared values. There is extensive evidence for the role of norms among survivors in emergencies (73). Some of these norms reflect pre-existing roles and rules that continue to operate, even in “extraordinary” events (74, 75). Other norms are constructed within the emergency itself (47, 76–78). Examples include the finding that men attempted to help women more than vice versa (i.e., gender role conformity) in the crowd crush at a concert by The Who (74), and the greater assistance offered to the elderly and infirm than the able-bodied in the Beverly Hills Supper Club fire (79).

#### Existing Relationships

The third kind of explanation for the evidence of survivors acting as responders is in terms of existing social relationships, which provide obligations (52) and motivations (80, 81) to stay with familiar others and to help them, even at risk to self. Social capital [e.g., (82)], the major framework used in policy for explaining sociality following disasters, emphasizes existing social bonds. Social capital refers to the ways that trust and reciprocity stemming from social networks can benefit people (83–85). There is extensive evidence for the role of social capital in disaster preparedness and community resilience (6, 86, 87). For example, to guard against the effects of the tsunami that hit Japan in 2011, neighbors with strong interpersonal connections provided mutual support to each other by helping to place sandbags in each other's houses (88).

### The Social Identity Approach

A fundamental idea of the social identity approach (18, 19) is that, as well as personal identities (“me,” my personality etc.), we each have social identities based on our group and category memberships (e.g., women, Manchester United supporters, lecturers). We have multiple group memberships and therefore we have multiple social identities. Social identity is defined as “that part of an individual's self-concept which derives from his [sic] knowledge of his [sic] membership of a social group (or groups) together with the emotional significance attached to that membership.” [(89), p. 69]. Each social identity is associated with a particular set of norms, values, interests, and emotions; and so when we define ourselves in terms of particular identities we strive to enact and express the associated norms, values, interests, and emotions. Different social identities each become relevant to us at different times and contexts, as a function of who else is present (19) and how they are behaving (including toward us) [For more details, see (90)].

#### Crowds and Group Psychology From the Social Identity Perspective

The crowd (including the crowd in a mass emergency) is a particular kind of social group. It is an *ad-hoc* group where people are face to face in a relatively unstructured situation. The social identity model of crowd behavior (91) suggests that in such crowds people don't lose identity [as classical crowd psychology suggested; (92)] but rather may shift from personal to shared social identity. The norms and values—the definitions of appropriate conduct—of that shared identity shape crowd behavior, even in extreme or violent events such as urban riots (93).

Not all crowds share identity, of course. Some are simply individuals present in the same physical location—such as in crowds of shoppers or crowds in transport hubs. But in those crowds where people are with others they see as having the same social identity as themselves, behavior becomes more intimate and perceptions and expectations become more aligned: people talk more, support each other, coordinate, expect agreement, share emotion; these factors often make the experience an enjoyable one (94).

Historically, crowd psychology theories have been preoccupied with violent crowds (92). A critical point made by the *elaborated* social identity model of crowd behavior [ESIM; (95, 96)] is that one cannot properly understand conflictual crowd behavior unless one takes *context* into account. Context has two aspects. First there is the *intergroup* context, which refers to the fact that crowd events often comprise two or more crowds (for example a protest crowd and the police), and that the relation between them can vary (in terms of both power and perceived legitimacy). Second, there is the *historical* context. For example, previous actions by the authorities might help explain crowd's hostile reaction to the authorities' present actions, even where the authorities themselves believe they are acting fairly.

While most of these insights arose from the study of conflictual crowd events, as we shall see some of their implications can be carried across to the psychology and management of mass emergencies. First, however, we show how the three types of process described above—emergent disaster communities, norms, and existing relationships—which have largely been expressed in sociological or interpersonal terms, can be developed through the framework of social identity.

#### Disaster Communities Are Based on Emergent Shared Social Identities

Research suggests that shared social identity among survivors is a crucial mediating mechanism between perceptions of common fate and supportive behavior in emergencies (71, 97). Thus, an interview study with 21 survivors of different kinds of emergencies (98) (including the sinking of the Jupiter, Hillsborough football stadium disaster, Accra [Ghana] football stadium crush, and the Bradford football stadium fire) found that reports of shared danger were more common for survivors that identified strongly than for those who identified weakly with others affected. Further, whereas most of those who identified strongly reported giving help, only a minority of people who identified less strongly reported giving help.

Stronger evidence for the same relationship came from a cross-sectional questionnaire survey of a representative sample of 1,240 survivors of an earthquake and tsunami that took place in Chile in 2010 (99). The study found that disaster exposure predicted common fate, and common fate predicted social identification with others affected by the disaster, but there was no direct pathway between common fate and helping behavior. Further, social identification with others affected by the disaster predicted both giving emotional social support and (indirectly, through expected support) participation in practical support activities for the whole community. Similarly, post-disaster surveys of adults and children affected by earthquakes in Italy found that common fate predicted shared identity (100) and that shared identity based on common distress predicted helping intentions (101).

As well as being evident in “sudden impact” emergencies, the same pattern has been found in the aftermath of a “rising tide” emergency. The city of York, UK, was hit by storm Eva in December, 2015, which resulted in the flooding of ~350 houses and 150 businesses. Analysis of interviews with residents conducted 2 months later identified various factors that enhanced perceptions of common fate (102). Directly and indirectly affected residents stated that they came to see themselves as sharing a community identity with others affected by the flood due to the similar experience of an adverse event, due to suffering from similar problems that followed the event such as looting, and as a result of common struggles against the lack of the necessary infrastructure. The emergent sense of community became the basis for the provision of social support. Residents reported providing others with practical support, such as helping to move furniture to higher levels, and emotional support, such as listening to others' needs and comforting them. The same relationship between common fate, emergent shared identity and social support was found in a predictive model, based on survey data from the same population (103).

**Figure 1:**
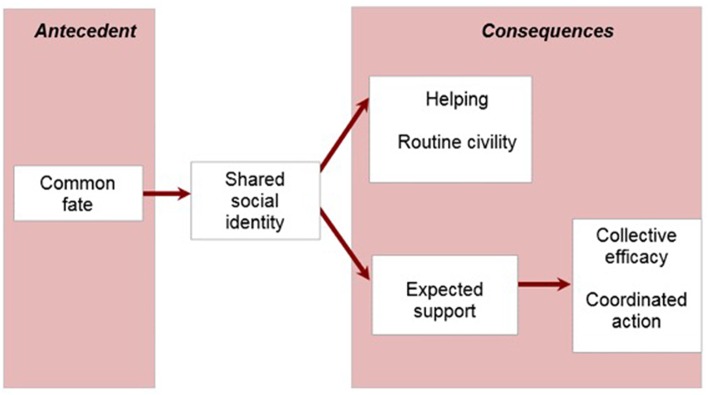
Social identity model of collective psychosocial resilience in emergent groups.

The above evidence together suggests that shared social identity is the psychological basis of disaster communities. This notion was expressed formally in *a social identity model of collective psychosocial resilience in emergent groups* (71, 97, 103, 104) (see Figure 1). Here, “collective resilience” refers to the way a shared identification allows groups of survivors to express and expect solidarity and cohesion, and thereby to coordinate and draw upon collective sources of support, to deal with adversity [(98), p. 502].

#### Social Norms Are Group Norms

The social identity approach suggests that social norms are *group* norms. As well as societal norms which are widely shared, different groups within a society each have different norms. Some group identities place a particular value on charity, solidarity or harmony (105), especially in particular identity-relevant contexts—for example Muslim pilgrims at Hajj (106). In the absence of such norms, salience of subgroup identities may lead to less solidarity following disasters toward those seen as outgroup members (107); but an ingroup norm of charity or solidarity would increase the help offered to these outgroup members. Some groups have more individualistic identities, according to which personal independence might be normative (108, 109). For example, group norms of individual “self-reliance” are associated with reduced participation in coordinated evacuations from hurricanes (110).

Further, commitment and conformity to particular group norms is a function of commitment to particular group identities (108). The comparative interview study described earlier (98) found that those who strongly identified with fellow survivors were more likely than others to report conforming to societal norms and the rules of their role (e.g., teacher). Possibly identification with a group defined only in terms of the emergency context leads to greater conformity to norms and role most relevant to the new group (71).

In relation to *emergent* norms, the Chile earthquake study described above (99) found that observing emotional and coordinated social support was associated with providing the same kinds of support. Importantly, the connection between observing others' supportive behavior and providing support oneself was stronger for those that identified with the category of other survivors. This analysis is in line with Reicher's (93) argument that, in ambiguous crowd events, people look to others as a guide to their own behavior insofar as these others are clearly a member of the individual's social group and as long as their behavior doesn't contradict existing group norms (111).

#### Existing Relationships Based on Shared Identities

Social capital approaches focus on networks of established relationships, whether interpersonal or as part of groups, as the basis of the trust and helping intentions required for supportive interventions among survivors in emergencies [e.g., (88)]. The social identity approach adds to this by suggesting that it is our *identification* with these groups and social categories that underlies trust and motivation to help.

For example, combining social capital and social identity perspectives, Helliwell and Barrington-Leigh (112) found an association between community belonging (their measure of social identity) and trust in one's neighbors. Group-based trust in strangers who are members of an existing ingroup social category has also been demonstrated experimentally (113) and has been shown to increase helping intentions in bystander intervention (114, 115), among those affected by a disaster [the 2011 Great East Japan Earthquake; (116)], and in terms of charitable donations to those affected by disasters (117, 118). More generally, for those with stressful jobs, identification with one's work-group has been shown to increase the social support needed to reduce stress (119).

The social identity approach has a number of implications for policy and practice, and hence is the basis for the recommendations provided in this article. Before this, however, we detail how the phenomenon of survivors as immediate responders has been addressed in recent UK guidance on emergency management.

## Assessment of Existing Guidance

In this section, we summarize the findings of three recent critical reviews of UK guidance on managing emergencies. We also update these reviews by showing how two recent pieces of research on social identity processes in emergencies—the first on the July 7th London bombings and the second on mass casualty decontamination for Chemical, Biological, Radiological, and Nuclear (CBRN) incidents—have begun to change the official guidance.

It is necessary to place the UK guidance in context by briefly describing the key events and trends that have shaped it and which have led to recognition of the role of the public as responders. Two trends stand out as shaping UK policy and practice in emergencies: the response to the threat of terrorism since 9/11; and the effects of climate change (in particular flooding).

### The UK Context: Threats and Mitigations

#### The Consequences of 9/11

Before the 9/11 attack on the World Trade Center, emergency planning was still informed by the experience of World War II (120). After 9/11, as well as increased securitization of everyday life, one noticeable development was that the term “resilience” became particularly prominent in policy discourse. The UK government sought to promote “resilience,” not only in response to an increase in terrorist attacks, but also for attacks that were qualitatively different from those of the past (including suicide bombers and CBRN incidents, and more recently marauding attackers and vehicle attacks).

#### Climate Change

Weather-related disasters are increasing (121) and climate change is having direct impacts including through flooding and heatwaves (122). For example, at the European level, temperature increases of 1.5–3.0 degrees Celsius are associated with increased flood risks in Central and Western Europe (123). The UK is among the countries most prone to flooding (124) and flooding is one of the major national risks in the UK in terms of both impact and likelihood [(125), p. 8]. Around 5 million people in 2.4 million properties in the UK face some risk of flooding, with 330,000 properties facing a significant risk. This number is projected to rise to between 630,000 and 1.2 million properties by 2080 due to the impacts of climate change (126).

#### The Civil Contingencies Act

In relation to both terrorism and floods, the increased threat, and the likelihood that there would not be enough professional responders immediately available for each incident, were factors that led the UK government to look increasingly to the capacities of the public. The Civil Contingencies Act [CCA; (126)] was the institutional response. The inclusion within this civil contingencies framework of a programme of “*community* resilience” was an explicit acknowledgment of the essential role of informal public collective response (127). A recent guidance document indicates the autonomy and agency envisaged in the public:

Community resilience is about empowering individuals, businesses and community groups to: take collective action to both increase their own resilience and that of others; come together to identify and support vulnerable individuals; take responsibility for the promotion of individual and business resilience (128).

### Analysis of the UK Guidance

In this section we examine how social psychological processes have been referred to in the guidance on emergency management, and the implications of these for policy and practice.

#### UK Emergency Management Guidance, From 9/11 to 2011

The analysis summarized here (2) covered publicly available event safety or civil contingencies preparedness non-statutory guidance produced in the UK. For the civil contingencies documents, the start date was 2001, the year of the attacks on the World Trade Center. For the health and safety documentation, this boundary was extended to 1999, since some documents had not been revised in that time. The end date was 2011.

A systematic internet search for open-access documents on Google using keywords, plus prior knowledge of certain key documents [such as the *Purple Guide*, (128)], led to a pool of 47 documents. Given the historical and practical importance of the “panic” concept in emergencies, we word-searched these documents for references to this word. Nine documents produced positive results. We were also interested in how informal public resilience was represented in the documents. To achieve this, the same nine documents selected for “panic,” plus those others in the pool (six) whose titles indicated that they were concerned with resilience in the public, were discourse analyzed. This entailed analyzing how a word or phrase was used, its relationship with other concepts, and its functions or effects. The 15 documents subjected to analysis are listed in Table 1.

**Table 1:** Documents included in analysis of guidance, 9/11 to 2011.

**Source**	**Year**	**Document**
Cabinet Office	2011	*Strategic National Framework on Community Resilience*
Cabinet Office	2011	*Preparing for Emergencies: Guide for communities*
Cabinet Office	2010	*Emergency response and recovery: Non statutory guidance accompanying the Civil Contingencies Act 2004*
Cabinet Office	2004	*Preparing for emergencies: What you need to know*
Cabinet Office	2011	UK Resilience: Communicating risk^*^
Cabinet Office	2006	Evacuation and Shelter Guidance^*^
Department of Health	2009a	*NHS Emergency planning guidance: Planning for the psychosocial and mental health care of people affected by major incidents and disasters: Interim national strategic guidance*
Department of Health	2009b	*Developing psychosocial resilience: How to cope in a crisis*.
Fire service	2003	National Guidance Document: Fire Service Mass Decontamination^*^
Health Protection Agency (HPA)	2008	CBRN Incidents: Clinical management and health protection^*^
Health and Safety Executive (HSE)	2000	Managing crowds safely^*^
Health and Safety Executive (HSE)	1999	Guide to Health safety and Welfare at Pop Concerts and Similar Events (Purple guide)^*^
Home Office	2004	The Decontamination of People Exposed to Chemical, Biological, Radiological or Nuclear (CBRN) Substances or Material^*^
London Emergency Services Liaison Panel (LESLP)	2007	Major incident procedure manual (7th edition)^*^
London Resilience Team (LRT)	2009	London regional resilience flu pandemic response plan^*^

* Contains references to “panic.”

*Table adapted from Drury et al. (2)*.

While the fact that “panic” was present in roughly a fifth of the documents sampled in the database of this study suggests it is only a minor feature, the nature of what is said, not just the quantity, is important. Characterizing behavior as “panic” provides a set of expectations and assumptions that are different from more neutral ascriptions (like “emergency egress,” “flight,” or “rapid evacuation,” for example). Some of the documents were explicit that it is collectives (especially crowds) that are vulnerable to “panic,” which was implied to be emotional in essence and so unreasonable. Some stated that, given this collective psychological vulnerability, care must be taken by those managing the emergency not to be open with information to the public (129). While some of the myths about “crowd panic” were openly challenged in some of the guidance, the existence and nature of “crowd panic” itself as a phenomenon remained unchallenged.

There was some reference to endogenous crowd resilience and to support among strangers in three of the guidance documents, in line with the evidence in the research literature. For example, in the Strategic National Framework on Community Resilience (130), one of the four types of community cited was a “community of circumstance”:

These communities are created when groups of people are affected by the same incident, such as a train crash. These groups of individuals are unlikely to have the same interests or come from the same geographical area but may form a community in the aftermath of an event. Although this sense of community may be temporary, some communities of circumstance grow and are sustained in the long-term following an emergency (p. 12).

Yet this explicit recognition of the crowd of survivors as responders was allocated only a small amount of space in the guidance, and most of the recommendations for communities referred to geographical communities.

In other places, however, even where the public and the community were depicted as having some resilient qualities—such as sociality and the ability to process information—they were not represented as equal with the emergency services and the other relevant formal organizations. Mostly, the public were treated as essentially a recipient of public “services.” Rendering the public as relatively passive and dependent functions to exclude the public from equal participation and renders the professional responders indispensable, which is inconsistent with the assumption of a capacity for community resilience and the need for such a capacity.

This position of relative passivity, and the separation of the public from those for whom (most of) these documents are addressed, is made explicit in the definitions of “Category 1” and “Category 2” responders in 2004 Civil Contingencies Act, neither of which includes “the public” (131). One level down from Category 2, the category “convergent volunteers” represents the acknowledgment that members of public might act as responders, but only subsumed under in the “Third Sector” (charities, voluntary organizations).

#### CBRN Mass Decontamination Guidance, 2002–2016

Decontamination is a procedure undertaken to remove contaminants from the skin of a potential casualty in the event of a CBRN incident, and may involve quarantine and showering. As the procedure involves removing clothes, in some circumstances decontamination those affected may perceive decontamination as more threatening than the CBRN incident itself. Evidence from incidents of decontamination suggests that effective communication is important in the management of incidents involving decontamination; failure to communicate effectively has resulted in increased public anxiety and reduced public compliance with decontamination (132, 133), which increases secondary contamination risk for those affected, receiving acute healthcare facilities, and for the wider public.

Carter and Amlôt (15) and Carter et al. (16) reviewed decontamination the guidance for emergency responders, identifying descriptions of public behavior and any recommendations on how emergency responders should manage the public. Searches were limited to documents on decontamination which were available in the open literature, and which were produced in English after the 9/11 attacks. From keyword searches on Google and knowledge of the field, 19 guidance documents which met the inclusion criteria were identified. These are listed in Table 2.

**Table 2:** CBRN mass decontamination guidance documents, 2002–2016 included in analysis.

**Source**	**Year**	**Document**
U.S Army Soldier and Biological Chemical Command	2002	*Guidelines for cold weather mass decontamination during a terrorist chemical agent incident*
Metropolitan Medical Response System	2003	Rapid access mass decontamination protocol^*^
New Dimension Regional Team	2003	National guidance document: Fire Service mass decontamination^*^
Home Office	2004	The decontamination of people exposed to chemical, biological, radiological, or nuclear (CBRN) substances or material (2nd edition)^*^
Governor's Office of Emergency Services	2006	Multi-casualty mass decontamination guidance for first responders^*^
State Government Victoria	2007	Decontamination guidance for hospitals^*^
Health Protection Agency	2008	*Generic Incident Management*
HM Government	2008	*Fire and Rescue Manual. Volume 2: Fire Service Operations. Incident command*
U.S Army Edgewood Chemical Biological Center	2009	Guidelines for mass casualty decontamination during a HAZMAT/ weapon of mass destruction incident (volume II)^*^
National Health Service	2010	*NHS emergency planning guidance: The ambulance service guidance on dealing with radiological incidents and emergencies*
US Army Chemical, Biological, Radiological, and Nuclear School	2011	Guidelines for military mass casualty decontamination operations during a domestic hazmat/ weapons of mass destruction incident^*^
Department for Communities and Local Government	2012	*Fire and Rescue Service: Operational guidance incidents involving hazardous materials*
NHS Scotland	2012	Guidance for hospitals on surface decontamination of self-presenting persons potentially exposed to hazardous chemical, biological, or radiological substances^*^
Edgewood Chemical Biological Center	2013	Guidelines for mass casualty decontamination during an HAZMAT/ weapon of mass destruction incident: Volumes I and II^*^
Harvard School of Public Health	2013	*Strategies for first receiver decontamination: A collection of tactics to assist hospitals address common challenges associated with all-hazards decontamination of patients*
Home Office	2013	Guidance for the United Kingdom emergency services on decontamination of people exposed to hazardous chemical, biological or radiological substances^*^
International Atomic Energy Agency	2013	*Public decontamination*
National Ambulance Resilience Unit	2014	*National Ambulance Service CBRNE/ HAZMAT guidance – OFFICIAL*
US Department of Homeland Security	2014	*Mass chemical exposure incident: National planning guidance for communities*

* Contains references to controlling the public

*Table adapted from Carter and Amlôt (15) and Carter et al. (16)*.

Frame analysis suggested that the guidance documents focused predominantly on the technical aspects involved in decontamination (e.g., developing and testing decontamination equipment), with only 10 of the 19 documents including any discussion of likely public experiences and behavior. Where likely public behavior was considered within the guidance, discredited assumptions such as disorder and mass panic were common. As a result, the guidance emphasized the importance of controlling members of the public (10 documents), rather than communicating with them. There was a lack of reflection in the guidance on the possibility that distress and resistance from the public might be a function of the way the responders were managing them, rather than due to the psychology of the crowd *per se*.

#### UK Guidance on Floods and Community Resilience, 2006–2016

Given the threat of floods and importance of the community resilience framework as a strategy for UK governments to deal with their impact, Ntontis et al. (17) analyzed how “community resilience” was represented in the guidance for this type of disaster. Again what was of interest were the ways that social groups and psychological elements were portrayed, and the implications of these for practitioners and policymakers.

Similar to the earlier reviews, search was limited to open sources. A keyword Google search and consultation with experts identified 71 relevant documents. After limiting the pool to guidance documents that were issued by a UK government agency or department, referred to floods as types of disasters, and explicitly referred to community resilience, 28 guidance documents remained that would be subject to discourse analysis—see Table 3^3^.

**Table 3:** Documents used in the analysis of guidance on community resilience and flooding, 2006–2016.

**Source**	**Year**	**Document**
Cabinet Office	2006	*Emergency Preparedness: Non-Statutory Guidance accompanying the Civil Contingencies Act 2004*
Cabinet Office	2011	*Strategic National Framework on Community Resilience*
Cabinet Office	2013	*Emergency Response and Recovery: Non-Statutory Guidance accompanying the Civil Contingencies Act 2004*
Cabinet Office	2013	*Expectations and Indicators of Good Practice Set for Category 1 and 2 Responders*
Cabinet Office	2013	*Responding to Emergencies. The UK Central Government Response. Concept of Operations*
Cabinet Office	2016	*Preparing for Emergencies: Guide for Communities*
Cabinet Office	2016	*Roles, Responsibilities and Partnerships to build Resilient Communities*
Cabinet Office	2016	*Steps for increasing Community Resilience*
Cabinet Office	2016	*The Context for Community Resilience*
Civil Contingencies Secretariat	2013	*The Role of Local Resilience Forums: A Reference Document*
Committee on Climate Change	2015	*Progress in Preparing for Climate Change. 2015 Report to Parliament*
Committee on Climate Change	2016	*UK Climate Change Risk Assessment 2017: Synthesis Report: Priorities for the Next 5 Years*
DEFRA	2014	*The National Flood Emergency Framework for England*
DEFRA	2015	*Flooding in England: Lead Government Department Plan*
Department of Health	2009	*NHS Emergency Planning Guidance: Planning for the Psychosocial and Mental Health Care of People affected by Major Incidents and Disasters: Interim National Strategic Guidance*
Environment Agency	2015	*Under the Weather. Improving Health, Wellbeing and Resilience in a Changing Climate*
Healthcare System Adaptation Report Working Group	2015	*Adaptation Report for the Healthcare System 2015*
HM Government	2013	*The National Adaptation Programme: Making the Country Resilient to a Changing Climate*
HM Government	2015	*Government response to the Committee on Climate Change: Progress on Meeting Carbon Budgets and Preparing for Climate Change Summary Document*
HM Government	2015	*Meeting Carbon Budgets−2015 Progress Report to Parliament: Government Response to the Seventh Annual Progress Report of the Committee on Climate Change*
HM Government	2016	*National Flood Resilience Review*
London Resilience Partnership	2014	*Communicating with the Public Framework v1*.
London Resilience Partnership	2015	*Strategic Flood Response Framework*
Department of Environment, Heritage & Local Government	2013	*A Framework for Major Emergency Management: Guidance Document 11: A Guide to Flood Emergencies*
NHS England	2014	*NHS England Emergency Preparedness, Resilience and Response (EPRR): Planning for the Management of Self-presenting Patients in Healthcare Settings*
Sustainable Development Unit	2014	*Adaptation to Climate Change Planning Guidance for Health and Social Care organizations*.
Sustainable Development Unit	2014	*Module: Healthy, Sustainable and Resilient Communities*
Sustainable Development Unit	2014	*Sustainable, Resilient, Healthy People & Places*

*Table adapted from Ntontis et al. (17)*.

The analysis suggested that community resilience is represented in official UK guidance on flooding at different levels of complexity. Simple construals treated resilience as merely the opposite of vulnerability, or as a static and reified element that can be developed (by external agencies), but no mechanisms or processes for its development were mentioned. There was also a pattern of circularity within some documents whereby the enhancement of community resilience was treated as a mechanism for community resilience.

In contrast, some documents referred to specific elements that can enhance community resilience, such as leadership, communication, and collaboration between communities and authorities, effective skill use, resource availability, participation in emergency planning, and veridical beliefs. As found in the earlier review (2), emergent communities were mentioned (2011) but it was mostly pre-existing, geographical communities that were the focus of community resilience programmes.

#### Recent Developments: Including the Role of Social Identity Processes in the Guidance

In this section, we bring the analysis of emergency management guidance documents up to date by describing some recent developments. Specifically, two sets of research studies have been important for changing the guidance to include the role of crowd members as responders based on (emergent) shared social identities: on the July 7th London Bombings and on CBRN decontamination.

##### The July 7th London bombings research in the guidance

The London bombings comprised three explosions on the London Underground and one on a London bus in rush hour on July 7th 2005. Fifty-six people were killed (including the bombers themselves), and over 700 were injured. Many survivors remained underground out of contact with the emergency services for a period of time. The research comprised interviews and an extensive corpus of secondary data, which together provided accounts from 90 survivors plus 56 witnesses (11, 134–136). Analysis found that most people were commuters and were among strangers, but help was common and was associated with shared social identity arising from common fate, as described in earlier examples (98, 99).

The NATO guidance on psychosocial care for people in emergencies and disasters (60) draws upon the evidence from the London bombings study and the concept of collective psychosocial resilience as part of the rationale for the Stepped Model of Care. This embodies the idea of building on survivors' psychosocial capacities for rapidly forming new social bonds (rather than assuming them to be ill or helpless). The guidance therefore recommends practical support, not psychiatric care, for most people affected by emergencies.

The key principles of the NATO guidance (and references to the London bombings research and the concept of social-identity based collective psychosocial resilience), also informed the Department of Health Emergency Preparedness Division's (2009) NHS Emergency Planning Guidance (137), as well as Department of Health/NHS guidance on pandemics (138), produced for staff. The London bombings study is also referred to in the Cabinet Office (2012) revision to Emergency Preparedness, chapter 7 on communicating with the public (139), where it serves as part of the rationale for providing information to survivors. The study's finding that solidarity is common also informs the National Risk Assessment, a guide on risk and emergencies, which is used by Local Resilience Forums across the UK to take into account the psychological and behavioral impacts of disasters when assessing risk.

Further, following the 2017 Manchester Arena bombing, in which 23 people were killed and 139 wounded by a suicide bomber as they left a pop concert, the Kerslake report (140) on emergency preparedness recommended greater “public first aid training” and “realistic contingencies for public involvement… within all incident zones” (p. 154–6), citing Cocking (11):

What the selfless actions of the multiple “zero” and first responders… highlighted so clearly, is that casualty care at, and evacuation from, incident sites should never be planned as something that only fully trained, and expensively equipped personnel should be relied upon to do [(140), p. 156].

This recommendation to upskill the public in first aid echoes recommendations that were made after the London bombings (14) and are reflected in a new programme currently being planned (141).

##### New CBRN decontamination research in the guidance

CBRN mass decontamination represents a particular set of operational problems, as described earlier. Compliance and successful decontamination is not easy to achieve. Further, any attempt at coercing the public would probably be unfeasible (due to the numbers involved) and would certainly be counterproductive, as it is likely to reduce the legitimacy of the operation in the eyes of the public and therefore escalate collective conflict (142).

What is required, therefore, is not only for members of the public to understand what is needed but to internalize the goals of the decontamination procedure so that they are motivated to engage and self-organize. Carter et al. (142) developed hypotheses on how to achieve these goals through harnessing social identity processes, based on research in related domains of crowd behavior. Thus, work based on the ESIM (95, 96) had shown that communication from police that respected football fans' identities, norms, and needs increased perceptions of the legitimacy of police behavior among these fans (143), as well as their identification with the police (144) and subsequent compliance with police advice (145). Based on this, a mass decontamination field exercise (146), an online visualization experiment (147), and a mass decontamination field experiment (148) each showed that when fire and rescue personnel explained the importance of decontamination and provided regular updates about their actions, this increased perceptions of the legitimacy of the procedure. In turn, this increased identification between emergency responders and members of the public which predicted reduced public anxiety (146, 147), reduced public confusion during the process (148), greater public compliance, and cooperation (146–149), and greater speed and efficiency of decontamination (148). In short, the form and content of the communication changed the relationship, rendering the professional responders as ingroup and therefore leading the public to be more accepting of, and engaged with, the decontamination procedure (which was now understood as “our public health procedure” rather than an imposition).

Changes to the guidance (internationally as well as in the UK) following this research include reference to the key role of communication (150) and to some of the recommendations by Carter et al. on legitimizing responder behaviors (151, 152). There have also been changes to UK Fire and Rescue Service (FRS) decontamination practice, which have led to enhanced performance, and changes to government thinking about CBRN mass casualty decontamination (153). In short, these changes represent a move from representing the public as an obstruction to seeing them as a partner in the decontamination process.

#### Progress on Inclusion of Social Psychology in Emergency Management Guidance

This review of the guidance is not comprehensive. However, it has included the major UK guidance documents produced since 9/11, and in particular on mitigations for a very important terrorism-related threat (CBRN incidents) and for one of the main risk factors in the UK (flooding). In recent years, there has been progress in including reference to social identity based recommendations in the guidance. However, this progress has been uneven and inconsistent. Specifically, more needs to be said about emergent disaster communities, their identities, and group norms. Research shows that even for disasters happening to geographical locations (in particular flooding) the focus on geographical communities is insufficient, for two reasons. First, there is often an unequal distribution of damage and distress, which means that not everyone in the geographical location suffers in the same way (72). Second, the people that see themselves psychologically as a community do not always correspond to the geographical community. For example, Travelers, who normally live separately from settled communities, came to be included in the York floods disaster community while other locals were excluded (102).

The recommendation to provide the public with information is now an orthodoxy across official emergency preparedness and response guidance (2). This is undoubtedly a positive development, as all contemporary theories of mass emergency behavior as well as health behavior models (154–156) would agree that people are meaning-seekers and makers; information provides them with efficacy, reduces their anxiety, and empowers them (8, 101). But there are still questions over whether this is applied in practice, as many organizations still communicate in code about emergencies (42, 63). A deeper understanding is also needed of the social-psychological functions of communication, and how it interacts with identity processes. These are some of the points addressed in our recommendations.

## Twelve Actionable Recommendations

The following 12 recommendations are derived from the research above, but also from dialogue with communities affected by disasters, and with professional groups and bodies involved in emergency response—in particular the live events and crowd safety industry, the CBRN community, the UK Fire and Rescue Service, the Department of Health, Public Health England, and the Civil Contingencies Secretariat. Some of these organizations are already making use of social identity principles in their practice. The recommendations are intended for all practitioners and policy-makers involved in emergency planning and response, including resilience officers; crowd safety managers, trainers, and stewards; and the emergency services. Taking seriously the notion that the public are often responders, we also make recommendations that are relevant for the public themselves. The section is divided into the three conventional phases of emergency and disaster management in the UK: preparedness, response and recovery.

## Preparedness Phase

### Emergency Planners and Responders Should Understand Group Psychology

Popular culture, mass media accounts, and some psychological textbooks contain many wrong and dangerous assumptions about mass emergency behavior based on discredited crowd psychology—not only “mass panic” but also “contagion,” “de-individuation,” “mob mentality,” and “stampede” (31, 34–36, 38–41, 157–159). We recommend, therefore, that those involved in emergency management prepare by developing their knowledge, with a critical perspective—which means appreciating that not all theories of group psychology are equal, that some are much better evidenced than others. Specifically, there are four fundamental lessons from research on group psychology which need to be understood.

The first lesson is that *panic (over-reaction) is rare in crowds in emergencies* (43–49). This means that collective over-reaction should not be assumed to be a default. This does not mean that people will not flee, or that some individuals won't overreact, but that fear and fleeing aren't necessarily panic and that panic is not a typical crowd phenomenon.

The second lesson is that *social support among survivors is common in emergencies* (52–56). Not all emergencies are characterized by support, and not everyone in those emergencies is supportive. But support is common enough that it is widely recognized as a regular pattern across emergency events, and should be expected.

The third lesson is more specific to the social identity approach and has to do with process. It is that *much of this social support is due to shared social identity* (71, 97). This points to the need to understand, reinforce, and work *with* (not against) social identity. This is the focus of the other recommendations below

The fourth lesson is that crowd behavior is a function of the perceived legitimacy of other groups' behavior. Therefore, responders should understand that *the way in which they manage an incident will impact on public behavior* (1, 96, 143–149, 160).

### Plan to Work With, Not Against, Group Norms in Emergencies

Given the importance of group norms in groups' perceptions and behaviors, and given that evidence shows that these norms are maintained in emergencies, managers and professional responders need to work with, not against, those norms. For example, several aspects of decontamination in a CBRN mass casualty incident—such as disrobing and showering in front of others—go against general societal norms, and are therefore likely to result in distress. Shared social identity with others affected, and the development of a shared norm around decontamination, may help to make these aspects of decontamination more acceptable. We discuss in the sections below how to facilitate such new group norms during an incident. The point for *preparedness* is first simply to be aware of and *recognize these norms* as a source of possible distress, and design the procedure so that responders are seen to do as much as possible to respect privacy.

For some groups, such as those from certain cultural or religious backgrounds (e.g., Muslim women), being asked to disrobe in front of others may be especially distressing (15, 161). Demonstrating that religious needs are respected results in increased trust in responders, and increased willingness to comply with treatment (162). Specifically, it is important to plan for such religious needs by provisions such as gendered showering (15), as well as working with local religious authorities, who can help to identify solutions and get community buy-in. Within this recommendation, therefore, is the requirement for those professionals working with the public to have sufficient *cultural competence* to recognize when some emergency management procedures are a problematic issue.

### Develop Evidence-Based, Pre-tested Communication Strategies

We recommend communication for all three phases of an emergency because communication can operate differently in each phase. It is widely agreed that information provision is important. However, for those involved in emergency management, in the preparedness phase communication should mean not just *providing information* but, crucially, *listening*.

#### Provide Pre-incident Information and Identify Trusted Messengers

Pre-incident information can be provided in textual form [e.g., “Run, hide, tell”; (163)]. It can also be provided in the form of drills to enhance procedural knowledge. For example, Fahy and Proulx (164) suggest that drills following the attack on the World Trade Center in 1993 meant that spontaneous evacuation was much quicker and more efficient on 9/11 than in the earlier incident. The use of both preparedness information whether as text or through drills is premised on the assumption that, even in stressful situations, people can still remember and process some information.

However, the relationship between source and public is crucial for determining whether information is trusted and internalized (9). In social identity terms, trust is a function of the perceived identity of the source in relation to that of the recipient. People are more persuaded by messages from fellow ingroup members than outgroup members (165–167), and especially by those seen as prototypical of their ingroup (168, 169). Therefore, those responsible for emergency preparedness need to prioritize *relationships*—and specifically shared social identity—with the community as part of their work of communicating—whether in relation to flood plans, what to do in a chemical incident, or general advice about a terrorist attack.

The first step in understanding and building relationships is *listening and learning*.

#### Listen to and Learn From at-Risk Communities

Communication in the form of listening is a key element in models of community resilience (6, 170), since it enables understanding of those areas where social support is needed, and increase the connectedness within and between communities. Put differently, listening and learning enables two things: (1) *knowing/understanding identities (and hence norms)* and (2) *relationship (i.e., shared identity) building*.

##### Know community identities, understand their norms

Listening allows the authorities and professional groups to recognize and understand the needs of the public and the particular community they are supporting, which is in line with a principle of Psychological First Aid [(171); see (172)]. In social identity terms, this means getting to know the values and norms—the *identity* (or different identities)—of the communities in question. This is the equivalent in research on public order policing to the recommendation to *educate* (oneself) (143).

Knowledge of group norms can shape particular risk management strategies. A relatively mundane example from the event safety industry illustrates the point. The moshing behavior of young people at music events might be interpreted by someone unfamiliar with it as uncontrolled fighting. However, more experienced observers understand that the practice has clear rules and limitations on physical interaction (173)—for example anyone who falls down is immediately picked up. Therefore, rather than intervention to try to prevent moshing (and potentially antagonize a crowd who expect to be able to dance in this way) crowd safety management might involve preserving a space for moshing as well as a separate space for other kinds of audience engagement with the music.

Knowing a community's identity and norms is also crucial to enhancing and creating shared identity with that community. For a positive relationship the identity needs to be properly recognized (174).

##### Build relationships during emergency planning: build shared social identity between communities and responders

There are many ways to build a relationship, and one important strategy is inclusion (14, 124, 175, 176). Inclusive practices, such as involving the public in planning, function to display the authorities' trust that the public can self-organize; it is the display of trust that can foster shared identity (177) and therefore encourage ownership and group efficacy around the emergency/resilience plan (122). Therefore, *include the public in resilience planning*.

### For Communities: Form a Community Group (103)

As we have seen, one of the basic effects of shared identity in a group is social support and hence group efficacy, as well as safety, health, and well-being benefits (20). This recommendation for community members—for example in areas prone to flooding—is that they form a group to develop preparedness, including sharing information. Forming a group is advised for example by the National Floods Forum (178), and hence is an established recommendation in relation to flooding (122). Increasing feelings of community control also seems to be associated with better mental health outcomes; it helps identify and resolve secondary stressors (82, 179); and it may help with communication with authorities. What the social identity approach adds is that these group identities should be meaningful and valued to people, so that they provide members with identification, pride, continuity, and efficacy (20). It can help for the group to have a name, a web-presence (e.g., a Facebook group), and other signifiers of identity. Responders can be invited to be part of these groups, though they will still need to be led by community members.

## Response Phase

### Prioritize Informative and Actionable Risk and Crisis Communication

Often, the advice given to the public both before and during an emergency is on emotions, or how to feel: “remain calm,” “don't panic” [e.g., (180)]. We are not aware of evidence that this kind of advice either reduces unnecessary anxiety or increases the efficacy people need in an emergency. Indeed, if people are already very anxious, this advice is probably not enough to change that. Moreover, if there is already mistrust between the public and the authorities, advice that there is nothing to worry about might itself increase public anxiety (3, 181). People require practical information; this will help them to make informed decisions (9), but will also meet their emotional needs and make them less distressed (171, 172). However, before we explain how to deliver this practical information, we must again address the issue of relationship-building.

#### Build Shared Identity Between the Public and Responders Through Providing Information

In the response phase, relationship-building must be done under time-pressure. Here we suggest that this can be done within and through the provision of practical information, in a single operation. We use here the example of CBRN mass casualty decontamination to show how this can be achieved effectively.

As described above, a programme of work led by Carter showed that effective communication by responders increased public compliance during these crucial life-saving procedures, enhancing the efficiency of the decontamination process, and potentially reducing fatalities (146–149). Importantly, while features of the communication (information on both the why and the how of decontamination showering; respect; perceived openness) predicted compliance and cooperation, they did so *indirectly*, through perceived legitimacy and shared identity; it was legitimacy and shared identity that directly predicted the adaptive outcomes of efficacy, reduced anxiety, compliance, and more efficient decontamination. The way information is provided is a communicative act, that can convey fairness and care. By conveying care around shared public health aims, communication legitimized the decontamination process in the eyes of the public, leading to shared social identification between crowd and responders. In short, while effective communication requires trust (165–167), communication strategies can also *build* trust by building shared identity.

In practical terms, then, these findings testify to the importance not only of scripting explicit instructions to the public, but also making much more use of “soft skills”—including eye-contact, mirroring, and using the public's own language (rather than official terminology)—as a crucial ingredient in creating shared identity. These elements are therefore necessary in the training and guidance for responders.

Carter et al.'s research led to four specific recommendations on public engagement, now included as part of UK FRS decontamination training: *(1) show respect for the public's needs* (which means *listening* for information on needs, as well as using knowledge obtained in the preparedness phase); *(2) be open and honest; (3) provide health-focused information* (why is the procedure necessary?); *(4) provide sufficient practical information* (15, 142, 160).

#### Use Human Voices Rather Than Bells and Sirens to Communicate

The logic of the argument that communication can build shared identity is that the mode of communication needs to be able to convey identity-information. There is already evidence that alarms in the form of bells are less effective in promoting evacuation than voice-based alarms and public address systems (63, 182). The social identity analysis adds to the argument for using voice-based alarms rather than bells and sirens, since the former but not the latter can invoke shared identity (e.g., reference to “we” and “us”).

#### Communicate What You Know (and What You Don't Know)

Responders should provide information to the public in a timely way, and should not wait until all information is known before initiating communication; if information is not known, this should be explained, and updates should be given when further information becomes available (160). Providing information on information (e.g., saying how what is known about an incident has been established by responders) is another way of building or maintaining a connection with the public.

### Do Not Undermine Shared Identity During the Response [(20) p. 128, (97)]

As we have seen (98, 99, 136), shared identity among survivors often arises from features of the event itself—when “all are in the same boat.” But professional groups' actions might (inadvertently) inhibit this process. For instance, on public transport addressing people as “customers” (rather than “passengers,” for example) stresses the money relationship which has been found to encourage individualized and selfish behavior (183). Addressing members of the public as separate individuals may make personal identities more salient and so undermine the natural processes toward shared identity (184).

### Use Language and Instructions to Facilitate Shared Identity

As well as consciously avoiding undermining shared identity, there are actions that can be taken to support, scaffold or facilitate the shared identity, both within the crowd and between the professionals and the crowd, in addition to those described above (section Build Shared Identity Between the Public and Responders Through Providing Information).

For shared identity *within* the crowd, communications with the crowd should refer to the relevant social category (e.g., “One Direction fans”), use collective nouns (e.g., “community”), or use the group's own name for itself (e.g., “metalhead”) to reinforce the collective identity (134). To create or enhance shared identity *between* the crowd and professionals, simple techniques include referring to “us” and “we” (rather than “you”) when addressing the public, and referring to common goals.

### Accommodate the Public Urge to Help (103, 140, 184, 185)

If survivors are “zero responders,” then this means a different way of thinking about them and their role. Survivors (and “convergers”) try to help (whether or not they have expertise) (24). This has functions for the broader disaster community: involvement builds unity and trust; and it can makes people feel better. But the key point of course is that it is often necessary for the public to respond, given the inability of sufficient responders to reach survivors in time (9, 12), which means that it should be accommodated where possible. Drawing on the situation in Israel, where there is institutional support for citizens as “active bystanders”, Adini (185) lists a number of specific functions that survivors can perform, including reporting of the event, reconnaissance, and even assistance with triage. In concrete terms, accommodating the inevitable public urge to help could involve setting up a system where survivor needs (e.g., “I need to house my dog somewhere while my house is pumped out”) can be matched with people with the relevant resources (e.g., “I have a big garden and can take in a friendly dog”). Local community groups could organize such a system, with local authority support^4^.

### Recognize and Work With “Group Prototypes” for Influence During an Incident

An example of a near-disaster from the live events industry shows how those managing safety can use knowledge of group identity to facilitate a safe outcome (187). The Big Beach Boutique II was an outdoor music event at which the number of people attending (250,000) far exceeded the number planned for (60,000), which led to a number of dangers. One of the risk factors included some people climbing up lamp posts in front of the stage. It was impossible, and in fact could have been counterproductive, to use threat and coercion to get them down, since the stewards and police were overwhelmed. Indeed, in the context of a free dance music event, orders from figures of authority would probably mean people doing the exact opposite. Instead, the crowd safety personnel asked the DJ to intervene. In social identity terms, the DJ was the ingroup prototype to the crowd, and so would function as a leader for those who shared the identity of dance music fans at the event. When he encouraged people to come down from the lighting rigs, they did so peacefully, cheered on by the crowd. Following this, no one else climbed a lighting rig for the rest of the event, suggesting that the intervention had served to instantiate a new norm.

## Recovery Phase

### Maintain Active Communication With Recovering Communities

In the recovery phase, as in preparedness, the recommendations on communication refer to both providing information and listening.

#### Keep Survivors and Families of Victims Informed

Timely and accurate information from the authorities has been identified as a key aspect of community resilience (6), and this is true in the post-disaster recovery period, when survivors and the families of victims often have many questions and require ongoing detailed advice and appropriate information that targets specific needs. This renders the maintenance of proper communication an imperative (139), and therefore again means it is necessary for the authorities to maintain shared identity, through the strategies indicated earlier.

#### Keep Listening to Recovering Communities, and Act on This Information

Communities suffering from a disaster have a range of different needs in the recovery period, and these needs can change over time. Therefore, authorities need to keep listening to understand these needs (188). We illustrate this with a recent example, the Grenfell Tower fire, which took place in June 2017 [(189), p. 1] and killed 72 people, with hundreds more moved out of their homes, traumatized, and distressed (179). After the event, there were many donations. However, one of the locals affected stated that:

resources have been thrown at people, but nobody actually listens and ask the community members what they wanted to have. People were being given massage, reiki treatment, or art therapy right after the event; however, they were found unnecessary because their basic needs were not assessed by anybody (interview, 2018).

The problem of “convergence” can manifest itself as donations that are unsuitable and which volunteers then have to spend valuable hours sorting through (24). This activity takes away their initiative by sapping their energy. In this case, what was needed was for the outside public to listen more closely to the needs and preferences of those affected and for the authorities to act as a filter for donations, based on understanding these needs.

### Keep the Disaster Community Alive

When the sense of common fate declines in the recovery phase, those affected no longer use the disaster to define themselves, and so shared identity also declines (103). Disaster communities also run out of energy and resources (72), and their agency is often undermined by interventions by the authorities anxious to restore top-down “command and control” (37, 72). Yet for disasters like floods, the continued problem of secondary stressors in the recovery phase (190)—stressors like chasing insurance, rebuilding one's home, continued dislocation—means that the support enabled by the disaster community is needed more than ever, and responders and authorities should facilitate this.

Yet research suggests that the emergent identities arising in disaster can be sustained strategically. Put differently, as well as arising from common fate in a relatively passive way, a shared identity can be consciously and deliberately invoked through various actions by community members.

Interviews 1 year after the York 2015 floods (Ntontis et al., unpublished) found that some people who experienced the floods organized a neighborhood meeting to celebrate and keep alive the sense of community that emerged during the disaster. There were also plans to organize a summer street party to celebrate the disaster community. Participants linked ongoing social support to the ongoing sense of community belonging they maintained through these events. Therefore, the recommendation is to *organize group events* (103). To the authorities, the recommendation is to *facilitate the maintenance of community groups*. This means *prioritizing supporting public autonomy over restoring “command and control.”*

The follow-up study of York flood-affected people found that communication between residents acted as a way of providing emotional support (103). But such communication also reinforced and sustained the flood identity that was the basis of such emotional support: when people met up and talked to each other, they talked about their common flood experience and self-definition. Therefore, the recommendation is that *members of disaster communities need to keep talking to each other* as they recover in the months after the disaster. Space and place are important in this. A regular place to meet, such as a café or social center, creates the opportunity for interaction, and helps concretize the group as an entity^5^.

### Mobilize Wider Solidarity

The UK government guidance on the recovery phase (191) defines it as the process of rebuilding, restoring and rehabilitating the community following an emergency, but also states that it is more than simply the replacement of what has been destroyed [(191) section 5.1.3]. A disaster often brings to light needs for change [(191) section 5.1.4]. Hazards become disasters through human (in)action (192), and so some disaster communities re-purpose themselves around a need for *justice* (193). Such needs may be met through organizing to lobby those in authority. Shared identity is the basis for such collective action (194), as it allows people to act as one (91).

Here again we use the example of the aftermath of the Grenfell Tower fire. In the aftermath of the fire, community members formed their own groups that provided psychosocial support in different ways using community spaces (195). Grenfell United was a group created by survivors and families of victims to meet their immediate needs, but also to campaign for “safe homes, justice, and change” (196, 197), reflecting the grievance that no one was prosecuted for the deaths and many of those affected were not suitably rehoused (198). They organized a regular “Silent Walk,” which took the form of a march with placards (like a protest) but in silence (as in a remembrance). The Silent Walk served to create a visible, tangible unity (199) and enabled people from around the country to show their support (200). Participants explicitly defined those affected by the disaster as broader than the 72 victims—since the flammable cladding and issues of safety for those in social housing affected a much wider group (201). This broad definition served to mobilize a wider psychological community for justice and made Grenfell into a national issue with which many people could identify.

These campaigning activities produced results for the affected people, including ensuring that Grenfell residents were represented as a part of the official enquiry (202, 203). Therefore, the recommendation to those affected by a disaster is to *mobilize wider solidarity beyond the affected community* (204).

## Discussion and Conclusions

Public health is politics, as a number of people have said, and the phenomenon of members of the public collectively acting as responders has a number of political dimensions. In the first place, “resilience” in the public could be a double-edged sword for those in authority. Ideally, informal collective resilience in emergencies and disasters would mean independence and empowerment in the public (6). Yet the more independent and empowered the public becomes, the greater the possibility that they develop aspirations the authorities didn't anticipate or wish for—including operating without need for the authorities. The emergence of living “cognitive alternatives” (18) in disaster communities was recognized in the earliest academic accounts of these communities, which referred to the way the mutual aid and democratization of relations within these events offered a glimpse of a new kind of society (37, 47).

A further political dimension is also a first caveat. The idea of public “resilience” can operate as an excuse for abnegating government responsibility: “because they're resilient, we don't need to provide support and resources for them.” In what Chandler (205) calls “resilience ethics,” the political responsibility of institutions is rearticulated as a need for the communities to become more “self-aware” of their own “life-choices,” including in relation to protecting themselves from environmental threats. In the age of austerity, cuts to services aligning with the supposed “big society” of public volunteers have left many people more vulnerable (206). Furthermore, as Kaniasty and Norris (72) argue, the effects of disasters are not distributed equally, so those expected to be resilient by acting as public responders may be those with least resources to do so. While shared social identity gives unstructured crowds the capacity to spontaneously self-organize, those in authority—emergency services, local authorities, local resilience forums, official event organizers, and safety advisory groups—have knowledge of the risks (likelihood and severity), the emergency plan, venues and locations, exit route capacities, and so on. They also have resources. Therefore, they still have responsibilities.

A second caveat is that, in many disasters, survivors are simply physically unable to help themselves let alone others (9, 74, 207). Our 12 recommendations, like the existing guidance based on community resilience principles, are for those emergency events where there is some scope for public response.

In summary, the scholarly literature on mass emergency behavior suggests that the public often act collectively as responders in these events. Social psychological research and theory is highly relevant in this, for it suggests that the emergent disaster communities, norms, and existing relationships that structure these spontaneous supportive actions can often be explained in terms of shared social identities. The importance of this for policy and practice is in terms of the implications that can then be drawn for the ways that authorities' actions can facilitate or inhibit capacities for informal public resilience, based on knowledge of social identity processes. The UK guidance since 9/11 has increasingly recognized this informal public resilience, though qualitatively and quantitatively there are residual accounts of public behavior as either pathological or passive, which conflict with the community resilience agenda. Nevertheless, in recent years social identity research, which offers an underpinning explanation of community resilience and other public responder behaviors, has more explicitly made its way into the guidance. Including our 12 recommendations within emergency preparedness, response and recovery guidance, as well as in training, will help professionals involved in emergency management to support public resilient behaviors and will help the public to develop and maintain their own capacity.

## Data Availability

The datasets analyzed for this study can be found in the UK Data Service SN: 5987, http://doi.org/10.5255/UKDA-SN-5987-1.

## Author Contributions

JD took primary responsibility for preparing the manuscript. All authors assisted with writing sections and with manuscript preparation. All authors approved the final manuscript for submission.

### Conflict of Interest Statement

The authors declare that the research was conducted in the absence of any commercial or financial relationships that could be construed as a potential conflict of interest.

## Footnotes

^1^The term “disaster myths” is standard in disaster sociology. However, we have placed it in quotation marks to sound a note of caution. For example, those numerous studies reporting an absence of mass panic do not in themselves falsify the mass panic concept; as with Popper's (23) example of the black swan, there may yet be counter-examples.

^2^We problematize the term “looting” in this context because it is often used to refer not only to opportunistic burglaries but also to survivors requisitioning goods from abandoned shops for survival (36, 37).

^3^Documents of this kind are often revised; here we focused on the most up to date version of each document.

^4^Israel provides many examples of institutional support for active bystanders in emergencies. Other countries can learn from Israel about how to facilitate the engagement of active bystanders. However, the present Review does not include more material from Israel for the reason that civil contingencies arrangements there do not easily carry over to other countries (186). Cole et al. (12) gives an example in which the Magon David (Israeli ambulance service) give out first aid kits to bystanders at mass casualty events. This works well in Israel as emergency first aid is embedded in the national curriculum and all adults are expected to do military service. In the UK, USA, and much of Europe, there is no equivalent national service and the culture is very different. Experiences of community resilience in Israel are framed very much in the context of the ongoing intergroup conflict that has been present since the creation of the state of Israel (and even before). Where a country is at war, it is much more likely that citizens will identify with the state and engage with state-organized civil contingencies measures, training, and so on (as well as the fact that all citizens learn these skills as part of national service). While part of the argument we make in this Review is that skills such as first aid should be supported in the ordinary public [as the Kerslake report (140) recommends, based on our work (11)], ongoing conflict and compulsory military service is not part of the UK context, and this is not likely to change in the near future.

^5^Some in emergency planning would say that communities need eventually to move on from the identities associated with the disaster, and that the decline in such identities is therefore part of the recovery process. However, there is an important distinction to be made here between different types of identities associated with disasters. Where there is a “victim” identity in which “outsiders cannot possibly understand” their suffering, the possibility of forming supportive relationships to meet needs and go forward becomes closed off. But there are also disaster communities whose “resilient” identities are defined broadly enough to enable supportive external alliances—as discussed in our final recommendation.
