# Corrigendum: Facilitating Collective Psychosocial Resilience in the Public in Emergencies: Twelve Recommendations Based on the Social Identity Approach

**DOI:** 10.3389/fpubh.2019.00181

**Published:** 2019-06-27

**Authors:** John Drury, Holly Carter, Chris Cocking, Evangelos Ntontis, Selin Tekin Guven, Richard Amlôt

**Affiliations:** ^1^School of Psychology, University of Sussex, Brighton, United Kingdom; ^2^Emergency Response Department Science and Technology, Health Protection Directorate, Public Health England, Salisbury, United Kingdom; ^3^School of Health Sciences, University of Brighton, Brighton, United Kingdom; ^4^School of Psychology, Politics, and Sociology, Canterbury Christ Church University, Canterbury, United Kingdom

**Keywords:** collective resilience, social identity, crowds, emergency, disaster, guidance

There is an error in the **Funding** statement. The correct number for “Economic and Social Research Council” is “RES-000-23-0446.”

Additionally, in the original article, we neglected to include the data-sets for empirical papers used in this review. A **Data Availability** Statement has therefore been added to the article text:

“The datasets analyzed for this study can be found in the UK Data Service SN: 5987, http://doi.org/10.5255/UKDA-SN-5987-1.”

Lastly, the number for footnote 5 was incorrectly positioned. It should be positioned at the last paragraph of subsection **Keep the Disaster Community Alive** and not in the last paragraph of the previous subsection. A correction has therefore been made to move the footnote to the correct position.

The authors apologize for these errors and state that they do not change the scientific conclusions of the article in any way. The original article has been updated.

